# Generation of an inducible dCas9-SAM human PSC line for endogenous gene activation

**DOI:** 10.3389/fcell.2024.1484955

**Published:** 2024-11-29

**Authors:** Paolo Petazzi, Francisco Gutierrez-Agüera, Heleia Roca-Ho, Julio Castaño, Clara Bueno, Niuska Alvarez, Lesley M. Forrester, Ana Sevilla, Antonella Fidanza, Pablo Menendez

**Affiliations:** ^1^ Josep Carreras Leukemia Research Institute, Campus Clinic-UB, Casanova 143, Barcelona, Spain; ^2^ Spanish Network for Advanced Cell Therapies (TERAV), Carlos III Health Institute, Barcelona, Spain; ^3^ Spanish Cancer Network (CIBERONC), Carlos III Health Institute, Barcelona, Spain; ^4^ Department of Cell Biology, Physiology, and Immunology, Faculty of Biology, Institute of Neuroscience, University of Barcelona, Barcelona, Spain; ^5^ Centre for Regenerative Medicine, Institute for Regeneration and Repair, Edinburgh Medical School, Biomedical Sciences, University of Edinburgh, Edinburgh, United Kingdom; ^6^ Institute of Biomedicine, University of Barcelona (IBUB), Barcelona, Spain; ^7^ Edinburgh Medical School, Biomedical Sciences, University of Edinburgh, Edinburgh, United Kingdom; ^8^ Department of Biomedicine, School of Medicine, Casanova 143, University of Barcelona, Barcelona, Spain; ^9^ Institució Catalana de Recerca I Estudis Avançats (ICREA), Barcelona, Spain

**Keywords:** human PSCs, ISAM, dead-Cas9 activation, gene expression, AAVS1 safe harbour locus

## Abstract

The CRISPR/Cas9 system has transformed genome editing by enabling precise modifications for diverse applications. Recent advancements, including base editing and prime editing, have expanded its utility beyond conventional gene knock-out and knock-in strategies. Additionally, several catalytically dead Cas9 (dCas9) proteins fused to distinct activation domains have been developed to modulate endogenous gene expression when directed to their regulatory regions by specific single-guide RNAs. Here, we report the development of the H9 human pluripotent stem cell (hPSC) line expressing an inducible dCas9-SAM activator (H9-iCas9.SAM), designed to activate transcription of endogenous genes. The H9-iCas9.SAM cells were generated through targeted integration of an inducible CRISPR/Cas9-based gene activator cassette into the AAVS1 “safe-harbour” locus. Molecular analyses confirmed precise and specific integration, ensuring minimal off-target effects. Functional characterization revealed that H9-iCas9.SAM cells retain pluripotency and display inducible endogenous gene activation upon doxycycline treatment. The versatility of H9-iCas9.SAM cells was demonstrated in directed *in vitro* differentiation assays, yielding neural stem cells (ectoderm), hematopoietic progenitor cells (mesoderm), and hepatocytes (endoderm). This underscores their potential in developmental biology studies and cell therapy applications. The engineered H9-iCas9.SAM line provides a robust platform for investigating gene function and advancing next-generation cell-based therapies.

## Introduction

Over the past decade, the CRISPR/Cas9 system has emerged as a powerful and versatile tool for genome editing. This technology relies on the Cas9 endonuclease, which is directed to a specific genomic site by a single-guide RNA (sgRNA). Initially exploited for the relative ease of generating insertions and deletions (indels) (Jinek et al., 2012), and thus gene knockouts, the system has since been used in conjunction with other effector protein domains to perform a wide range of functions, including: removal or deposition of epigenetic marks (Liu et al., 2016), cell imaging (Ma et al., 2016), and gene activation or silencing (Chavez et al., 2016; Yeo et al., 2018) among others. Regarding the latter, mutations in residues involved in DNA catalysis have generated catalytically dead Cas9 (dCas9) proteins that lack nuclease activity while preserving DNA binding (Mali et al., 2013; Dominguez et al., 2016). When fused to effector/activation domains such as VP64 and p65 and directed to regulatory regions of a genomic locus using sgRNAs, these nuclease-null, dCas9 variants have been shown to modulate endogenous gene expression (Mali et al., 2013; Gilbert et al., 2013; Maeder et al., 2013; Qi et al., 2013). More recently, CRISPR/Cas9 variants have been used to facilitate gene editing without the need for double-strand breaks (DSBs) or donor DNA templates and without relying on the homology-directed repair (HDR) mechanism. Base editors and prime editors allow users to introduce point mutations and small indels by employing fusion proteins such as deaminase, which catalyses nucleotide conversion (Gaudelli et al., 2017; Komor et al., 2016), and reverse transcriptase (Anzalone et al., 2019). A very recent development is the Cas9-PiggyBac fusion, which uses a hyperactive version of the PiggyBac transposase to insert larger payloads (Pallarès-Masmitjà et al., 2021).

All these advancements in gene editing are crucial in the fields of advanced cell therapies and regenerative medicine, which primarily involve the transplantation of a patient with tissue or cells from a donor or from the own patient. The potential use of gene-edited patient-derived cells has opened a myriad of new possibilities for untreatable monogenic diseases, particularly for *ex-vivo* cell therapy, where the patient’s cells are harvested and engineered *ex vivo* before reinfusion. In addition, human pluripotent stem cells (hPSCs), including both embryonic stem cells (ESCs) and induced pluripotent stem cells (iPSCs), have the potential to generate any tissue or cell type *in vitro* (Protze et al., 2019), thus representing an ideal foundation for developmental biology studies, drug testing and cell therapy applications.

Here, we report the generation and characterization of a hPSC line (H9) with a targeted integration of an inducible dCas9.SAM activator into the AAVS1 “safe-harbour” locus (H9-iCas9.SAM). Engineered H9-iCas9.SAM cells allow for single or multiplex transcriptional activation of endogenous genes, thus representing a robust platform for investigating gene function and developing next-generation cell-based therapies.

## Materials and methods

### hPSC culture

The hESC line H9 was obtained from WiCell (Madison, WI, United States), and cells were maintained on Matrigel (BD Biosciences, San Diego, CA)-coated plates and fed daily with mouse embryo fibroblast (MEF)-conditioned medium supplemented with 8 ng/mL basic fibroblast growth factor (Miltenyi, Bergisch Gladbach, Germany), as previously described by our group (Bueno et al., 2013; Romero-Moya et al., 2017). H9 cells were passaged weekly by dissociation with 0.05% trypsin (Thermo Fisher Scientific, Waltham, MA). Approval for hPSC work was obtained from the ISCIII-Comisión Nacional de Garantías (26/2013).

### Nucleofection and selection of H9-iCas9.SAM cells

To provide the optimal repair template for our engineered H9 cells, we used 1 µg of the linearized iCas9.SAM plasmid (Addgene #211495). We then prepared ribonucleoprotein (RNP) complexes by mixing equimolar amounts (50 pmol) of sgRNAs (listed in Supplementary Table S1) and dCas9 protein (IDT, Coralville, IO). Finally, 2 × 10^5^ H9 cells (2.2 × 10^7^ cells/mL) were electroporated, together with the iSAM cassette and Cas9 RNPs, using the Neon system (Invitrogen, Carlsbad, CA) with the following settings: 3 consecutive pulses of 1400 V and 5 ms pulse width. Cells were immediately seeded into one well of a six-well plate coated with Matrigel and were selected with geneticin (100 μg/mL) starting 24 h later.

### Southern blotting

Genomic DNA was isolated using the QIAamp DNA Mini Kit (Qiagen, Leiden, Germany). Ten µg of DNA were digested with BglII (New England Biolabs, Ipswich, MA), separated on a 0.8% agarose gel, and transferred to a Hybond nylon membrane (RPN303B; Amersham Biosciences, Amersham, United Kingdom). Membranes were hybridized with DIG-dUTP-labeled probes. Probes were detected using a 1:5,000 dilution of AP-conjugated DIG-antibody (Roche Diagnostics, Basal, Switzerland) with CDP-Star (Sigma-Aldrich, St. Louis, MO) as a substrate for chemiluminescence. The probe was synthesized by PCR with the PCR DIG Probe Synthesis Kit (Roche Diagnostics) using plasmid DNA as a template. Primers used for probes are detailed in Supplementary Table S1.

### sgRNA delivery

For sgRNA delivery, the sgRNA (MS2) _puro (Addgene #73795) backbone was Golden Gate cloned with all the guide variants according to an established protocol (Konermann et al., 2014). *ASCL1*, *NEUROD1* and *CXCR4* sgRNA sequences depicted in Supplementary Table S1 were taken from (Chavez et al., 2016).

### Virus production

A second-generation lentiviral production system was used to produce viral particles in HEK293T cells. The psPAX2 packaging plasmid, pMD2.G envelope plasmid and the lentiviral transfer vector were co-transfected using polyethyleneimine (Polysciences, Warrington, PA), as described (Prieto et al., 2016). Virus-containing supernatants were harvested 48–72 h after transfection, concentrated by ultracentrifugation and tittered in HEK293T cells. For transduction, H9 cells were passaged 24–48 h before exposure to viral supernatants (multiplicity of infection of 10). H9-iCas9.SAM infected cells, were selected for sgRNA integration with Puromycin (0.3 μg/mL).

### Quantitative real-time PCR

Total RNA was extracted with the Maxwell RSC simplyRNA Cells Kit (Promega, Madison, WI, United States). Reverse transcription was performed with 1 μg of RNA using SuperScript III and random hexamer primers (Thermo Fisher Scientific). cDNA was diluted 1:4 and 1 μL was used for each 10 μL reaction. Real-time PCR was performed with the PowerUp SYBR Green master mix (Thermo Fisher Scientific) in triplicate on the Bio-Rad CFX384 real-time platform (Hercules, CA). All primer pairs were designed with Primer-BLAST software and validated by running a standard curve with serial dilutions of cDNA to guarantee correct amplification of primer pairs. *GAPDH* or *RPL19* were used as housekeeping genes. Supplementary Table S1 lists the sequences of all primers and sgRNAs used in this study.

### Flow cytometry

Supplementary Table S2 shows the antibodies used for flow cytometry (TRA-1-60-BV510, TRA-1-81-APC, SSEA-4-V450, Nestin-APC, CD31-BV510, CD34-PECy7, CD43-FITC, CD45-APC, unconjugated SOX17 and human Serum Albumin-APC). For staining, 200,000 cells were resuspended in 200 μL of PBS+2% fetal bovine serum (FBS) and the corresponding antibodies at dilutions depicted on Supplementary Table S2, for 20 min at 4°C. Cells were then washed twice with PBS 1X and acquired on a FACSCanto II flow cytometer equipped with FACSDiva analysis software version 8.0 (Becton Dickinson, San Jose, CA, United States).

### Teratoma formation assay

Undifferentiated H9-iCas9.SAM cultures at 80%–90% confluence were collected by enzymatic dissociation with collagenase IV, and 2 × 10^6^ cells were re-suspended and injected in a volume of 250 μL of Dulbecco’s modified Eagle’s medium (DMEM) and 50 μL of Matrigel subcutaneously in both flanks of 6- to 12-week-old NOD-Cg-Prkdc^scid^ Il2rg^tm1Wjl^/SzJ (NSG) mice (The Jackson Laboratory) housed under pathogen-free conditions in the animal facility of the Barcelona Biomedical Research Park (Gutierrez-Aranda et al., 2010; Petazzi et al., 2020). Mice were euthanized when tumours reached 1 cm^3^ diameter. Teratomas were removed, fixed overnight in paraformaldehyde-containing solution, embedded in paraffin, sectioned, and stained with hematoxylin and eosin to assess the presence of cells deriving from the three germ layers. Animal experimentation protocols were approved by the Animal Care Committee of the Barcelona Scientific Research Park (HRH-17-0015).

### Neural precursor differentiation

Neural progenitors were differentiated using the PSC neural induction medium (NIM; Thermo Fisher Scientific, #A1647801). H9-iCas9.SAM cells were dissociated to a single cell suspension and 3 × 10^5^ cells were seeded into a Matrigel-coated 6-well plate. NIM was replaced every other day. Neural stem cells (NSCs) were passed once weekly from day 7 following manufacturer’s specifications. Differentiation assays were performed in parallel with and without supplementation of 2 μg/mL of doxycycline.

### Hematopoietic differentiation

Undifferentiated H9-iCas9.SAM cells were treated with collagenase IV for 5 min and gently scraped off from the plate. The embryoid bodies (EBs) were transferred to low-attachment plates and incubated overnight in medium composed of KnockOut-DMEM (Thermo Fisher Scientific) supplemented with 20% non-heat-inactivated FBS for hESCs (Biowest, Nuaillé, France), 1X Glutamax (Thermo Fisher Scientific), 0.1 mM nonessential amino acids, and 0.1 mM β-mercaptoethanol. The medium was replenished on the next day and was supplemented with bone morphogenetic protein 4 (BMP-4) (50 ng/mL), FMS-related tyrosine kinase 3 ligand (Flt-3L) (300 ng/mL), stem cell factor (SCF) (300 ng/mL), interleukin-3 (IL-3) (10 ng/mL), interleukin-6 (IL-6) (10 ng/mL) and granulocyte-colony stimulating factor (G-CSF) (50 ng/mL), with medium changes every 3–4 days (Bueno et al., 2012; 2013; Gutierrez-Agüera et al., 2021). All growth factors and cytokines were from R&D Systems (Minneapolis, MN, United States). Differentiation assays were performed in parallel with and without supplementation of 2 μg/mL of doxycycline.

### Hepatocyte differentiation

For hepatic differentiation, H9-iCas9.SAM cells were first guided into definitive endoderm (DE) in RPMI-1640 medium supplemented with 1% nonessential amino acids, 1% B27 supplement without vitamin A, 2 mM L-glutamine and 50 units/mL penicillin/streptomycin (all from Gibco-Invitrogen). Differentiation was initiated (day 0) when the cell density reached 3.2–5.2 × 10^5^ cells/cm^2^. On day 1, the medium was changed and supplemented with 100 ng/mL Activin A, 50 ng/mL BMP-4 and 6.45 μM CHIR99021 (all from R&D Systems). From day 2 to day 5, cells were supplemented with only 100 ng/mL of Activin A. After DE differentiation, H9-iCas9.SAM cells were differentiated to hepatocyte-like cells following the Cellartis Hepatocyte Differentiation protocol (Cellartis Hepatocyte Diff Kit; Takara Bio Europe AB, Gothenburg, Sweden; Y30050). Hepatocyte Progenitor Medium was used for medium changes on days 9 and 11 of differentiation. Finally, on day 14, cells were cultured with Hepatocyte Maturation Medium Base (3A) supplemented with Hepatocyte Maturation Medium Supplement (3B) according to manufacturer’s specification. Differentiation assays were performed in parallel with and without supplementation of 2 μg/mL of doxycycline.

### Main findings

#### Generation and characterization of the H9-iCas9.SAM cells

**FIGURE 1:**
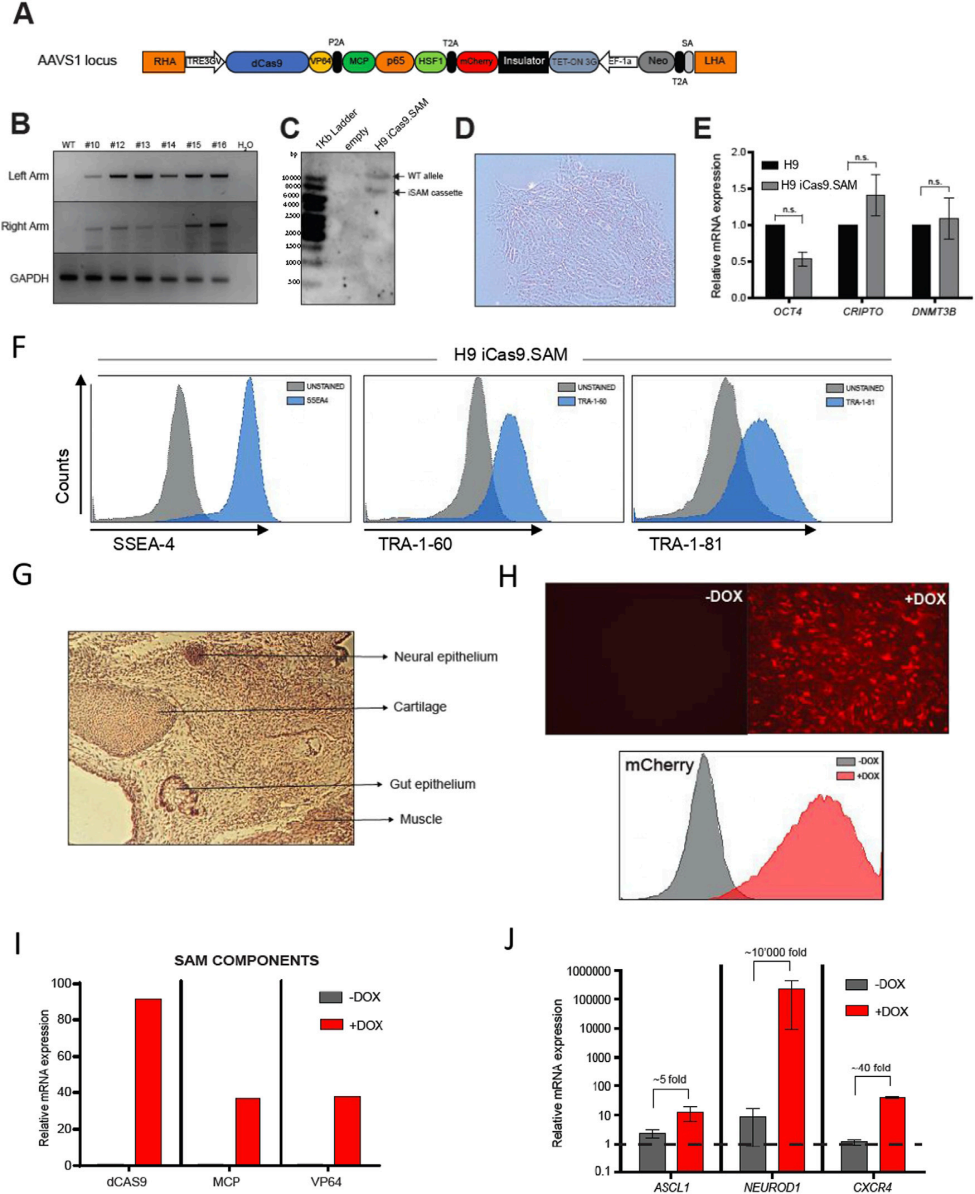
Generation and characterization of the hPSC line H9 with targeted integration of an inducible dCas9.SAM activator into the AAVS1 safe harbour locus (H9-iCas9.SAM). **(A)** Schematic representation of the donor vector used for insertion of the iSAM cassette into the AAVS1 locus. Right homology arm (RHA); left homology arm (LHA); splice acceptor (SA); dCas9-VP64-MCP-p65-HSF1, SAM components cassette; neomycin (neo); self-cleaving 2A peptide (P/T2A); inducible promoter (TRE3GV); constitutive promoter (EF1α), transactivating protein (Tet-ON 3G); mCherry reporter. **(B)** Targeted integration analysis of the cassette into the AAVS1 locus analysed by boundary PCR at the LHA and RHA using primers specific for the 5ʹ and 3ʹ integration junctions. Several H9-iCas9.SAM clones are shown. Full-length gels are presented in Supplementary Material. **(C)** Homologous recombination confirmed by Southern blotting after restriction enzyme digestion of genomic DNA from the neomycin resistant H9-iCas9.SAM clone #16 using a 5′ internal probe. Both wild-type (WT) and iSAM bands are shown, confirming heterozygosis. Full-length gel is presented in Supplementary Material. **(D)** Representative phase-contrast image of H9-iCas9.SAM clone #16 showing undifferentiated hESC-like morphology. **(E)** qRT-PCR of the indicated pluripotency genes in H9 WT and H9-iCas9.SAM (n = 2). **(F)** H9-iCas9.SAM cultures retain expression of the cell surface pluripotency markers TRA-1-60, TRA-1-81 and SSEA4. Insets represent unstained cells. **(G)** H9-iCas9.SAM cells successfully form teratomas in NSG mice. Arrows point to different cellular structures representing the three germ layers. **(H)** Robust expression of mCherry upon 2 μg/mL doxycycline induction for 2 days of H9-iCas9.SAM cultures. *Upper panels*, fluorescence microscopy images. *Bottom panel*, flow cytometry analysis. **(I)** qRT-PCR showing robust upregulation of the SAM components upon doxycycline induction of H9-iCas9.SAM cells. **(J)** H9-iCas9.SAM cells transfected with sgRNAs targeting *ASCL1, NEUROD1* and *CXCR4* show robust transcriptional activation upon induction with doxycycline.

To establish a doxycycline-inducible hPSC line expressing the SAM activator upon stimulation, we targeted the AAVS1 locus. This genomic locus harbours the *PPP1R12C* gene and has been extensively described as a safe harbour for transgene expression (Castaño et al., 2017; Smith et al., 2008). Knock-in of our iCas9.SAM cassette was performed by generating a DSB using CRISPR/Cas9 and providing the HDR machinery with a plasmid template for introduction into the AAVS1 locus (Figure 1A). H9 hPSCs were electroporated with the Cas9/sgRNA complex alongside the iSAM donor cassette, followed by selection with geneticin. Surviving colonies were then expanded. To confirm the successful integration of the iSAM cassette into the AAVS1 locus, we designed a specific genomic PCR assay to screen multiple clones simultaneously, identifying those exhibiting correct integration of both homology arms (Figure 1B). Subsequently, clone #16, designated H9-iCas9.SAM, was chosen for further characterization. To validate the exclusive integration of the iSAM cassette into the safe harbour, we conducted Southern blot analysis. As depicted in Figure 1C, a single copy of iSAM was detected at the AAVS1 locus (8.5 kb band), with the other allele remaining wild type (12.5 kb band), indicating heterozygous integration. We further validated the specificity of our sgRNA against the AAVS1 locus with an off-target analysis. The top 6 *in silico* predicted candidates (*RNF4, RHOT2, FAIM2, RPL8, BTNL8,* and *MYBL2*) were sequenced in H9-iCas9.SAM cells and consistently found to be unaltered (data available at https://github.com/anasevilla/H9iSAM/blob/main/H9%20iSAM_Sequencing%20off%20targets-20240308T162235Z-001.zip).

The H9-iCas9.SAM line exhibited typical hESC-like morphology (Figure 1D) and retained the expression of the pluripotency-associated transcription factors *OCT4*, *CRIPTO* and *DNMT3B* (Figure 1E), and the expression of the pluripotency-associated cell surface markers TRA-1-60, TRA-1-81 and SSEA4 (Figure 1F). To confirm that the transgene insertion did not compromise the pluripotency of H9-iCas9.SAM cells, we performed a teratoma assay in NSG mice. Careful anatomopathological analysis of the hPSC-derived tissues revealed the presence of derivatives from all three germ layers (Figure 1G). To confirm the specific induction of the activation iSAM cassette, we treated H9-iCas9.SAM cells with 2 μg/mL of doxycycline. After 48 h, more than 95% of the cells expressed mCherry, confirming that our system was successfully induced (Figure 1H). Furthermore, a robust expression of the individual components of the iSAM activation system (dCas9, VP64 and MCP) was observed upon doxycycline treatment (Figure 1I).

Lastly, we evaluated the capacity of H9-iCas9.SAM cells to activate endogenous gene expression by confirming effective targeting of multiple loci. To achieve this, we simultaneously activated three endogenous genes associated with various germ layers: *ASCL1*, representative of both ectoderm and mesoderm (Gao et al., 2016; Woods et al., 2022); *NEUROD1*, representative of ectoderm (Matsuda-Ito et al., 2022); and *CXCR4*, representative of mesoderm and endoderm (Kowalski et al., 2019; Nelson et al., 2008).This was accomplished by co-transfecting H9-iCas9.SAM cells with distinct sgRNAs directed at the regulatory regions of each gene. Upon induction with doxycycline, all three endogenous genes (ASCL1, NEUROD1, and CXCR4) were successfully activated by the dCas9-SAM activator, although at varying levels (see Figure 1J). Previous studies, including ours, have demonstrated an inverse correlation between the basal gene expression state and the level of activation of a given gene with dCas9-based systems (Chavez et al., 2016; Fidanza et al., 2017; Petazzi et al., 2020).

### Doxycycline-induced iSAM cassette remains expressed upon hPSC differentiation

The potential of hPSCs in developmental biology studies and cell therapy applications hinges on their capacity to differentiate into various cell types. To explore this potential, we employed H9-iCas9.SAM cells and subjected them to directed differentiation protocols aimed at generating neural stem cells (NSCs), hematopoietic cells, definitive endoderm (DE), and hepatocytes, representing ectoderm, mesoderm, and endoderm, respectively.

**FIGURE 2:**
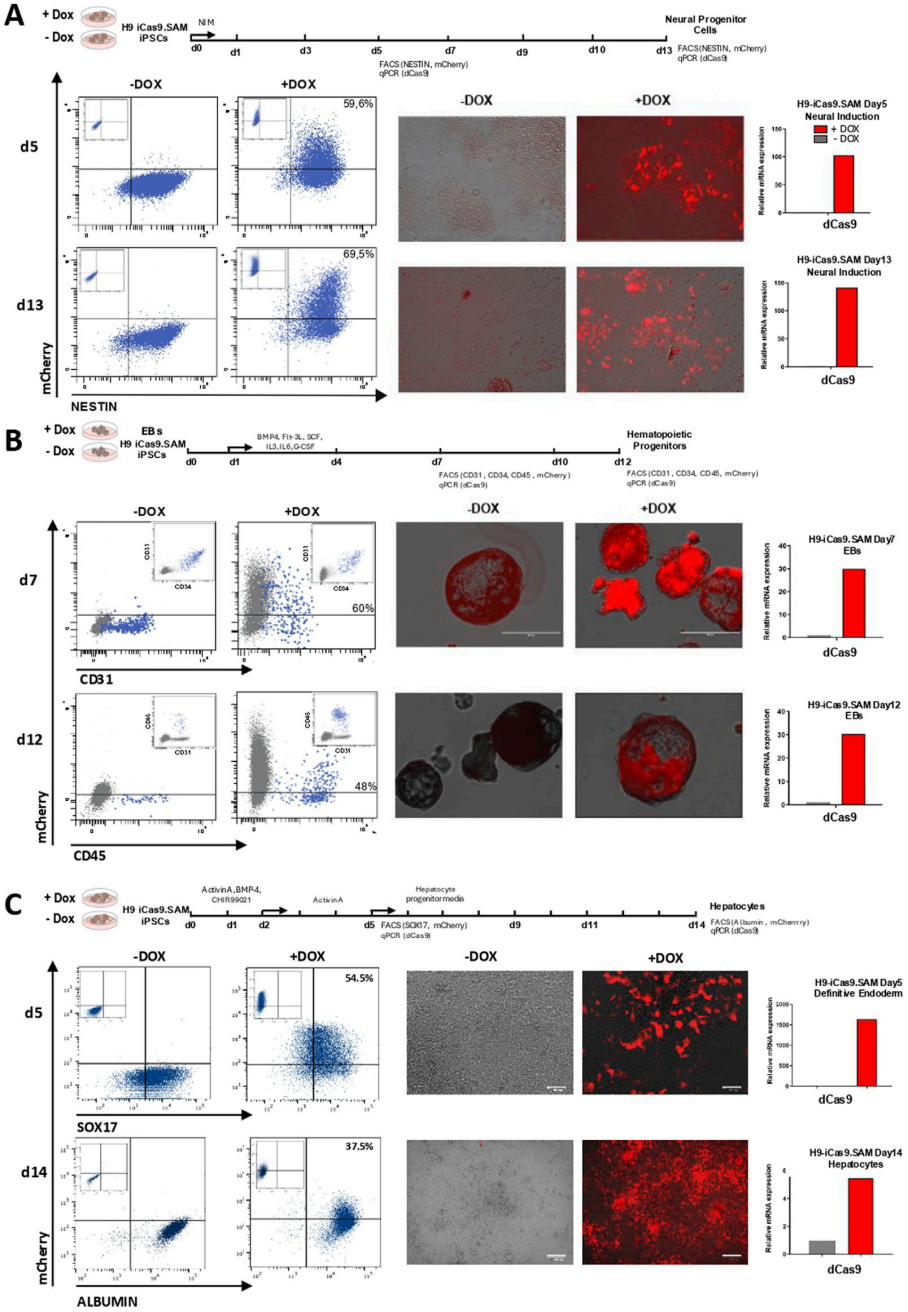
Doxycycline-induced iSAM cassette remains expressed upon hPSC differentiation. **(A)** Neural differentiation analysed at days 5 and 13 of differentiation. **(B)** Hematopoietic differentiation analysed at days 7 and 12 of EB development. **(C)** Hepatocyte differentiation analysed at days 5 and 14 of differentiation. *Left panels,* Flow cytometry analysis showing mCherry expression upon doxycycline treatment in the differentiating neural (Nestin+, shown in blue), hematopoietic (CD31^+^CD34^+^ or CD45^+^, shown in blue) and definitive endoderm (SOX17+, shown in blue) and hepatocytes (albumin+, shown in blue), respectively. *Middle panels*, fluorescence microscopy images confirming mCherry expression upon doxycycline induction at each respective stage of the specific directed differentiation protocol. Scale bar 100 µm for definitive endoderm and hepatocyte cells. *Right panels*, qRT-PCR showing robust expression of dCas9 upon doxycycline induction at each respective stage of the specific directed differentiation protocol.

NSCs, known as multipotent progenitors of neurons and glia (Shi et al., 2012), offer valuable insights into neuronal physiology and disease modeling. Using a commercial kit, we successfully cultured Nestin + NSCs within just 5 days of differentiation, with over 90% of the cells expressing Nestin (Figure 2A, left panels). Following induction with doxycycline, approximately 60% of these NSCs expressed mCherry (Figure 2A, left and middle panels), confirming the effective induction of the iSAM cassette. A specific 100-fold upregulation of the dCas9 (iSAM) cassette in the doxycycline treatment further validated the successful induction (Figure 2A, right panels). Remarkably, H9-iCas9.SAM-derived NSCs were sustained by day 13 of differentiation, exhibiting consistent levels of iSAM activation and even higher proportions (70%) of mCherry + Nestin + NSCs (Figure 2A, bottom panels).

We next induced hematopoietic differentiation from H9-iCas9.SAM cells using an EB-based differentiation protocol. During *in vitro* hematopoietic development, hPSCs are first specified into hematoendothelial progenitors (HEPs: CD34^+^CD31^+^), which then progress to CD45^+^ hematopoietic cells (Chadwick et al., 2003; Gutierrez-Agüera et al., 2021). CD34^+^CD31^+^ HEPs and CD45^+^ cells were consistently detectable at day 7 and day 12, respectively (Figure 2B, left panel). Following doxycycline treatment, sustained and robust expression of mCherry (Figure 2B, left and middle panels) and dCas9 (Figure 2B, right panels) was observed in both CD34^+^CD31^+^ HEPs and CD45^+^ cells, confirming successful iSAM activation upon mesodermal differentiation.

Finally, H9-iCas9-SAM cells were differentiated into hepatocytes. We first induced differentiation into SOX17^+^ DE and observed >90% SOX17^+^ cells by day 5, 55% of them expressing mCherry specifically in the doxycycline treatment (Figure 2C, left and middle panels). A massive upregulation of the dCas9 was observed in the doxycycline treatment confirming the successful activation of the iSAM (Figure 2C, right panels). Further differentiation of SOX17^+^ DE into hepatocytes rendered >98% of Albumin^+^ cells by day 14 of differentiation with a slightly lower but sustained expression of mCherry and dCas9 in ∼40% of the Albumin^+^ cells, confirming successful iSAM activation after endodermal specification (Figure 2C, bottom panels).

## Discussion

Human PSCs, which include both embryonic stem cells (ESCs) and induced pluripotent stem cells (iPSCs), are powerful tools in regenerative medicine due to their ability to differentiate into various cell types representing all three germ layers: ectoderm, mesoderm, and endoderm (JA et al., 1998; Takahashi et al., 2007). Leveraging the differentiation potential of these cells offers significant opportunities for tissue engineering, disease modeling, and drug discovery.

In this study, we generated an H9-iCas9.SAM-edited hPSC line through the targeted integration of an inducible CRISPR/Cas9-based gene activator cassette into the AAVS1 locus. This H9-iCas9.SAM hPSC line retains pluripotency both *in vitro* and *in vivo* and exhibits inducible endogenous gene activation.

Temporal regulation of gene expression is critical for mimicking the complex signaling cascades and activating transcriptional programs underlying embryonic development. dCas9-based systems allow for the precise control of gene expression dynamics throughout the differentiation process. The H9-iCas9.SAM hPSC line enables the simultaneous induction of lineage-specific genes upon doxycycline induction in human cells, which can be further multiplexed through guides directed to specific regulatory regions for multiple human lineage specific therapeutic applications. By modulating the activity of dCas9-based effectors in a spatiotemporal manner, gene expression patterns can be fine-tuned to recapitulate the reprogramming process (Sokka et al., 2022; Weltner et al., 2018), as well as *in vivo* differentiation processes more faithfully. For instance, CRISPR-mediated transcriptional activation (CRISPRa) reprogrammed cells have already been tested *in vivo* as a treatment for myocardial infarction (MI). This study used dCas9-SAM to reprogramme mouse tail-tip fibroblasts into CRISPR-induced cardiovascular progenitor cells (ciCPCs) by upregulating specific cardiac transcription factors, including GATA4, NKX2–5 and TBX5 (Jiang et al., 2022). Following myocardial infarction in mice, ciCPCs were injected into the heart between the infarct and border zone. Results showed a reduced adverse remodeling such as left ventricular dilation, reduced scar formation, and increased ejection fraction compared to controls. Similarly, another study used dCas9-VP64 to upregulate GATA4, MEF2C, NKX2–5, HAND2 and TNNT2 in rat cardiosphere-derived cells showing a significant improvement in the left ventricular ejection fraction (Sano et al., 2022).This CRISPR activation with the SAM system has also been applied for the differentiation of mouse embryonic fibroblasts into functional induced hepatocyte-like cells by either combining the expression of two transcription factors, GATA4 and FOXA3. AAV6-based delivery of the CRISPRa SAM system effectively induced the hepatic reprogramming from fibroblasts in mice with live fibrosis showing a significant reduction of the liver fibrosis after 8 weeks of induction (Li et al., 2024).

By generating disease-relevant cell types from patient-derived iPSCs and modulating gene expression to mimic disease states, researchers have elucidated the pathophysiological mechanisms underlying various disorders in cardiomyocytes and T lymphocytes (Mandegar et al., 2016). Both loss-of-function of tumor suppressor genes and gain-of-function of several oncogenes are key to the onset and progression of various types of cancer. Our iCas9.SAM hPSC line allows for in-depth exploration of early differentiation and oncogenesis mechanisms through controlled gene activation studies in cell populations previously differentiated from PSCs. Likewise, it represents a unique model for drug screening studies aimed at identifying specific activators or inhibitors of gene expression (Menendez et al., 2006). Moreover, dCas9-based systems like the one we have developed can be utilized in high-throughput screening assays to identify potential therapeutic targets and drug candidates for regenerative medicine applications (Kearns et al., 2015; Polstein and Gersbach, 2015). Additionally, organoids—complex 3D structures derived from stem cells—have emerged as valuable models for studying human development and diseases such as colorectal cancer (Matano et al., 2015; Drost and Clevers, 2017; Yui et al., 2012).

Genomic engineering allows for the creation of organoids with specific genetic mutations associated with diseases, enabling the study of disease mechanisms and the screening of potential therapeutics (Geurts et al., 2023; Lancaster et al., 2013; Madhavan et al., 2018). Combining CRISPR with tissue-specific derived organoids presents a promising pathway for advancing the clinical translation of genome engineering. While CRISPR–Cas9 offers high editing efficiency, safety concerns—especially in *in vivo* applications—continue to pose challenges. Tissue-specific organoids help address these concerns by allowing gene repair to be performed *ex vivo* with thorough off-target analyses, as demonstrated with our iCa9.SAM H9 line. These organoids can also be rapidly expanded, and once a corrected clone is deemed safe, it can be scaled and transplanted back into the patient to restore function to the affected tissue. However, tissue-specific transplantation protocols are currently limited, with much of the foundational work focused on systems like the mouse intestinal organoids (Drost and Clevers, 2017; Yui et al., 2012). Looking forward, advances in tissue engineering, together with genomic editing, hold the potential to significantly advance the development of functional tissues and organs suitable for transplantation (Atala et al., 2012).

## Conclusion

Taken together, we present the development and characterization of an H9 hPSC line expressing an inducible dCas9-SAM activator, integrated into the AAVS1 safe-harbour locus. This engineered H9-iCas9.SAM PSC line enables inducible activation of endogenous genes upon doxycycline treatment and serves as a robust platform for studying gene function and advancing next-generation cell-based therapies.

## Data Availability

The datasets presented in this study can be found in online repositories at https://github.com/anasevilla/H9iSAM/blob/main/H9%20iSAM_Sequencing%20off%20targets-20240308T162235Z-001.zip and in the Supplementary Material.

## Supplementary material

SUPPLEMENTARY TABLE S1 List of oligonucleotides (primers) used in the present study.

SUPPLEMENTARY TABLE S2 List of antibodies used in the present study.

## Abbreviations

CRISPRa, CRISPR-mediated transcriptional activationdCas9: catalytically dead Cas9; DSBs, double-strand breaks; EBs, embryoid bodies; ESCs, embryonic stem cells; FBS, fetal bovine serum; hPSC, human pluripotent stem cell; HDR, homology-directed repair; MEF, mouse embryo fibroblasts; NIM, neural induction medium; NSCs, Neural stem cells; sgRNA; single-guide RNA.
