# Supplementary material for: Generation of an inducible dCas9-SAM human PSC line for endogenous gene activation
Source: Front Cell Dev Biol. 2024 Nov 29;12:1484955. doi: 10.3389/fcell.2024.1484955 (PMC11638181; doi:10.3389/fcell.2024.1484955)
Supplement: Supplementary file 1 [file Table1.pdf]

Table S1. List of oligonucleotides (primers) used in the present study.

**Primer sequences for RT-PCR**	
AAVS1_iSAM_ left_F	GCCTCTGGCCCACTGTTTCC
AAVS1_iSAM_ left_R	CCACGTCACCGCATGTTAGAAG
AAVS1_iSAM_ right_F	CTCGAGTGAAGACGAAAGGGC
AAVS1_iSAM_right_R	CCAGCCCACCCCAATGCTC
GAPDH_F	GCACCGTCAAGGCTGAGAAC
GAPDH_R	AGGGATCTCGCTCCTGGAA
RPL19_F	GCGGAAGGGTACAGCCAAT
RPL19_R	AGCAGCCGGCGCAAAATCC
OCT4_F	GGGTTTTTGGGATTAAGTTCTTCA
OCT4_R	GCCCCCACCCTTTGTGTT
CRIPTO_F	CGGAACTGTGAGCACGATGT
CRIPTO_R	GGGCAGCCAGGTGTCATG
DNMT3B_F	GCTCACAGGGCCCGATACTT
DNMT3B_R	GCAGTCCTGCAGCTCGAGTTTA
Cas9_F	AGCACGTGGCACAGATCCTGG
Cas9_R	GGAAATCCTTCCGGAAATCGG
VP64_F	AAAAGAGGAAGGTGGCGGCC
VP64_R	CGTCACTGCCGAGCATGTCG
MCP_F	AAGGTGACATGCAGCGTCAGG
MCP_R	CCATGTTCAGGTAGGACCTCC
ASCL1_F	GAACTGATGCGCTGCAAACG
ASCL1_R	TGACCAACTTGACGCGGTT
NEUROD1_F	GGATGACGATCAAAAGCCCAA
NEUROD1_R	GCGTCTTAGAATAGCAAGGCA
CXCR4_F	ACTACACCGAGGAAATGGGCT
CXCR4_R	CCCACAATGCCAGTTAAGAAGA
***g*RNA sequences for CRISPR/Cas9 gene editing and activation**	
AAVS1 T2	GGGGCCACTAGGGACAGGAT
ASCL1	CGGGAGAAAGGAACGGGAGG
NEUROD1	AGGGGAGCGGTTGTCGGAGG
CXCR4	CCGACCACCCGCAAACAGCA
**Primers for Southern blot probe**	
AAVS1_probe_F	AGGTTCCGTCTTCCTCCACT
AAVS1_probe_R	GTCCAGGCAAAGAAAGCAAG
**Primer sequences for off-target gene amplification and sequencing**	
RPL8_OT_F	GCAGGCAGTTCTAGAAGCCA
RPL8_OT_R	CCTTAGTTATCTGGATTTCCAGAAC
BTNL8_OT_F	TAGGAGTCTTGGTGGTGTTCAT
BTNL8_OT_R	ATATCGTGGCACCTGGCTAC
FAIM2_OT_F	AGGCTCGTCCCATCCTTTTG
FAIM2_OT_R	CACATCCCCATTTGCTCCCT
MYBL2_OT_F	CTCCTGGCCCCTCTTAGACT
MYBL2_OT_R	GCAGTCGGAGGAAGTGACAA
RHOT2_OT_F	TGTTACTGGGCGAGGGTAGG
RHOT2_OT_R	CTACGGCCGCTACCTGAGTA
RNF4_OT_F	CAGACCGTGACTCCCGAAAA
RNF4_OT_R	GTCAGCGGGGAACAAAAACC
