# Supplementary material for: Generation of an inducible dCas9-SAM human PSC line for endogenous gene activation
Source: Front Cell Dev Biol. 2024 Nov 29;12:1484955. doi: 10.3389/fcell.2024.1484955 (PMC11638181; doi:10.3389/fcell.2024.1484955)
Supplement: Supplementary file 2 [file Table2.pdf]

Table S2. List of antibodies used in the present study.

Antibody	Cat Number	Dilution	Provider
Human TRA-1-60	560173	1/10	BD Biosciences
Human TRA-1-81	09-0011	1/250	Stemgent
Human SSEA-4	560219	1/10	BD Biosciences
Human Nestin-APC	IC1259A	1/10	R&D systems
Human CD31-BV510	563454	1/10	BD Biosciences
Human CD34-PECy7	348811	1/10	BD Biosciences
Human CD43-FITC	560978	1/10	BD Biosciences
Human CD45-APC	555485	1/10	BD Biosciences
Human SOX17	AF1924	1/20	Bio-techne R&D Systems
Human Serum Albumin-APC	IC1455A	1/20	Bio-techne R&D Systems
