# Supplementary figures and images for: Generation of an inducible dCas9-SAM human PSC line for endogenous gene activation
Source: Front Cell Dev Biol. 2024 Nov 29;12:1484955. doi: 10.3389/fcell.2024.1484955 (PMC11638181; doi:10.3389/fcell.2024.1484955)

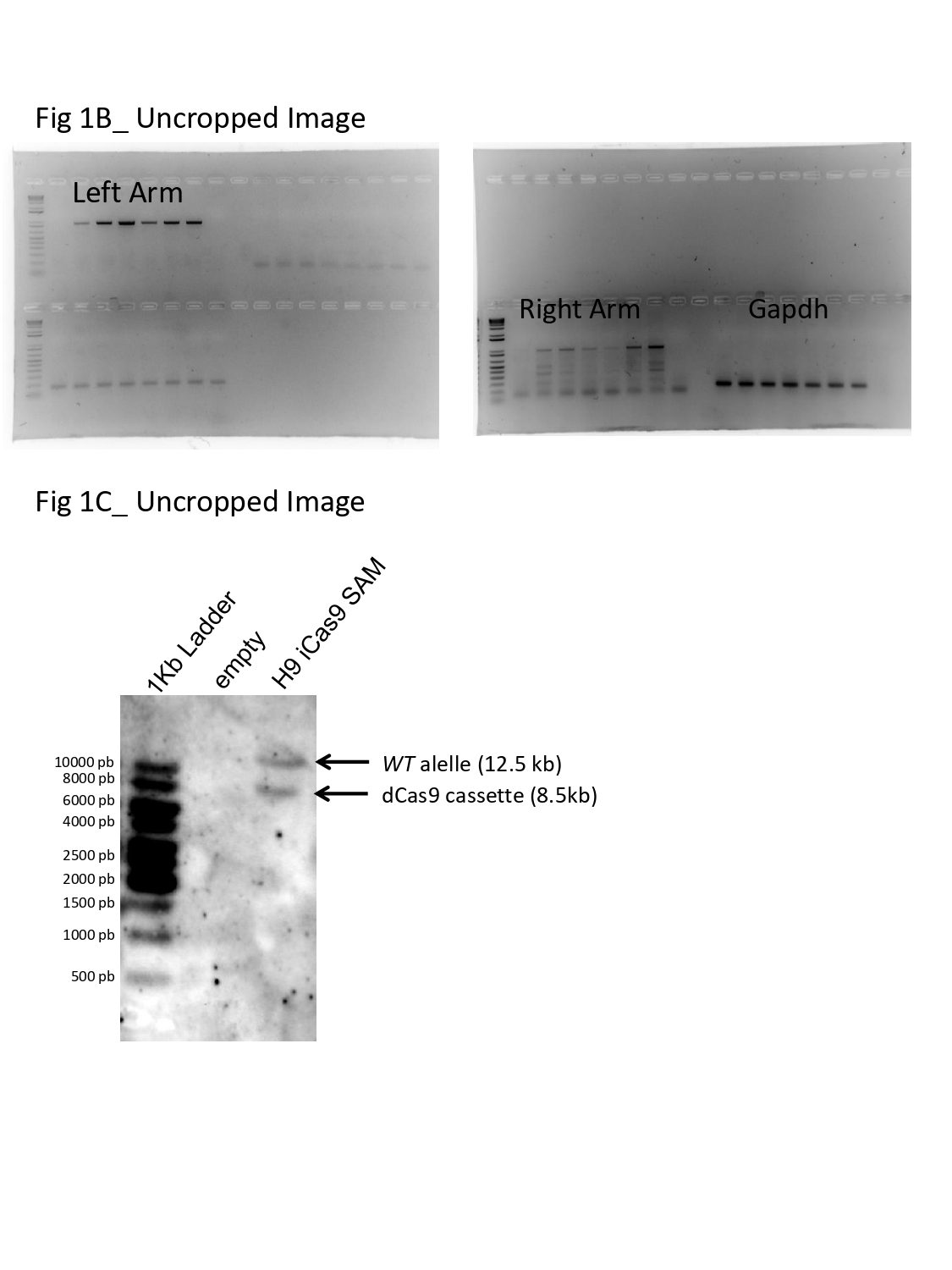

Supplement: Supplementary file 3 [file Image1.jpeg]

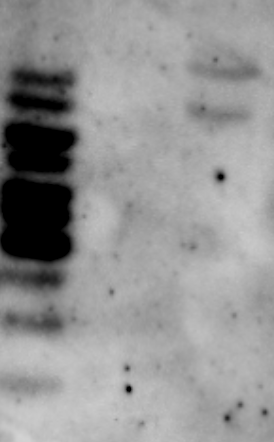

Supplement: Supplementary file 4 [file DataSheet1.zip › southern 2 (1).tif]

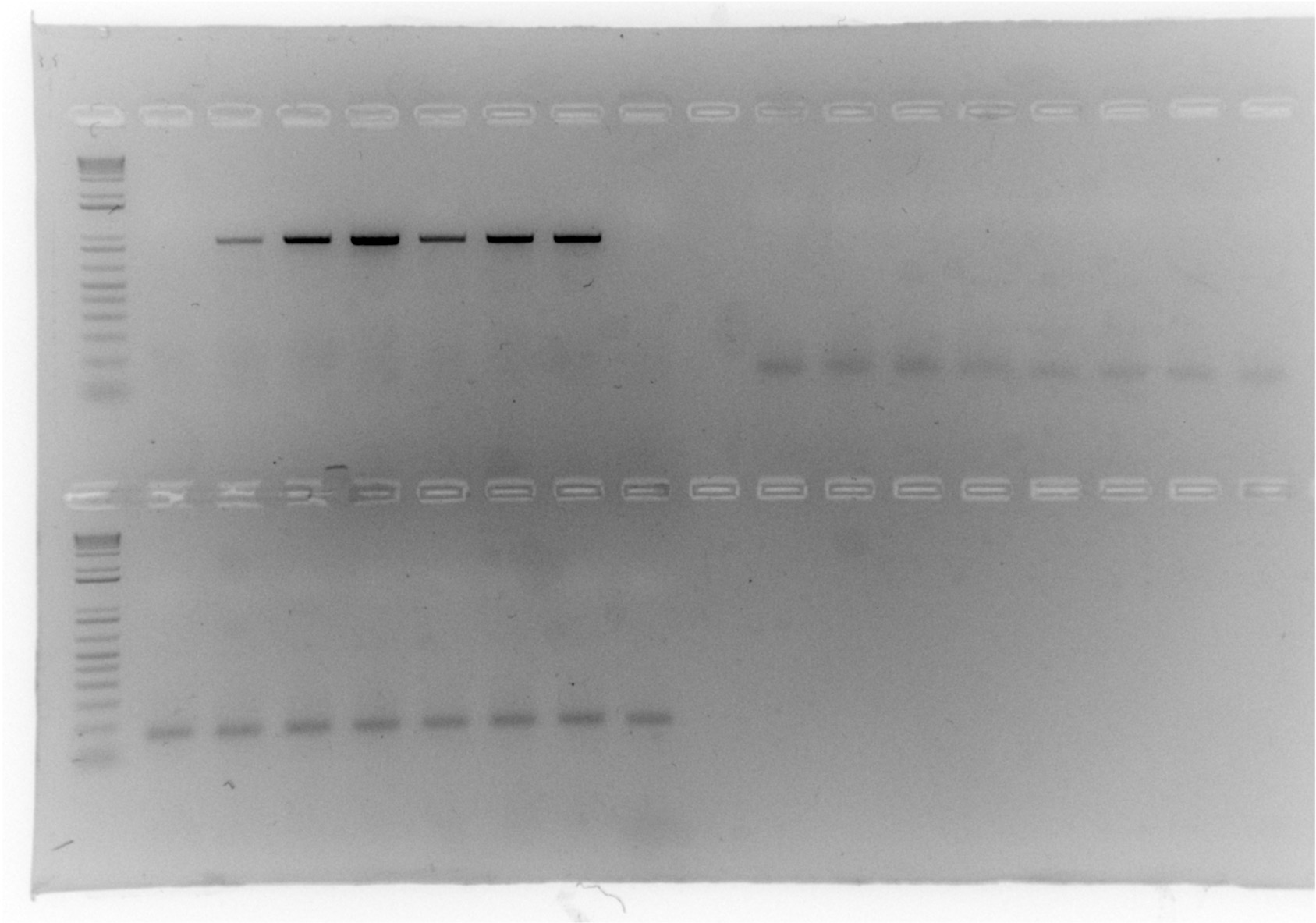

Supplement: Supplementary file 4 [file DataSheet1.zip › Left arm.tif]

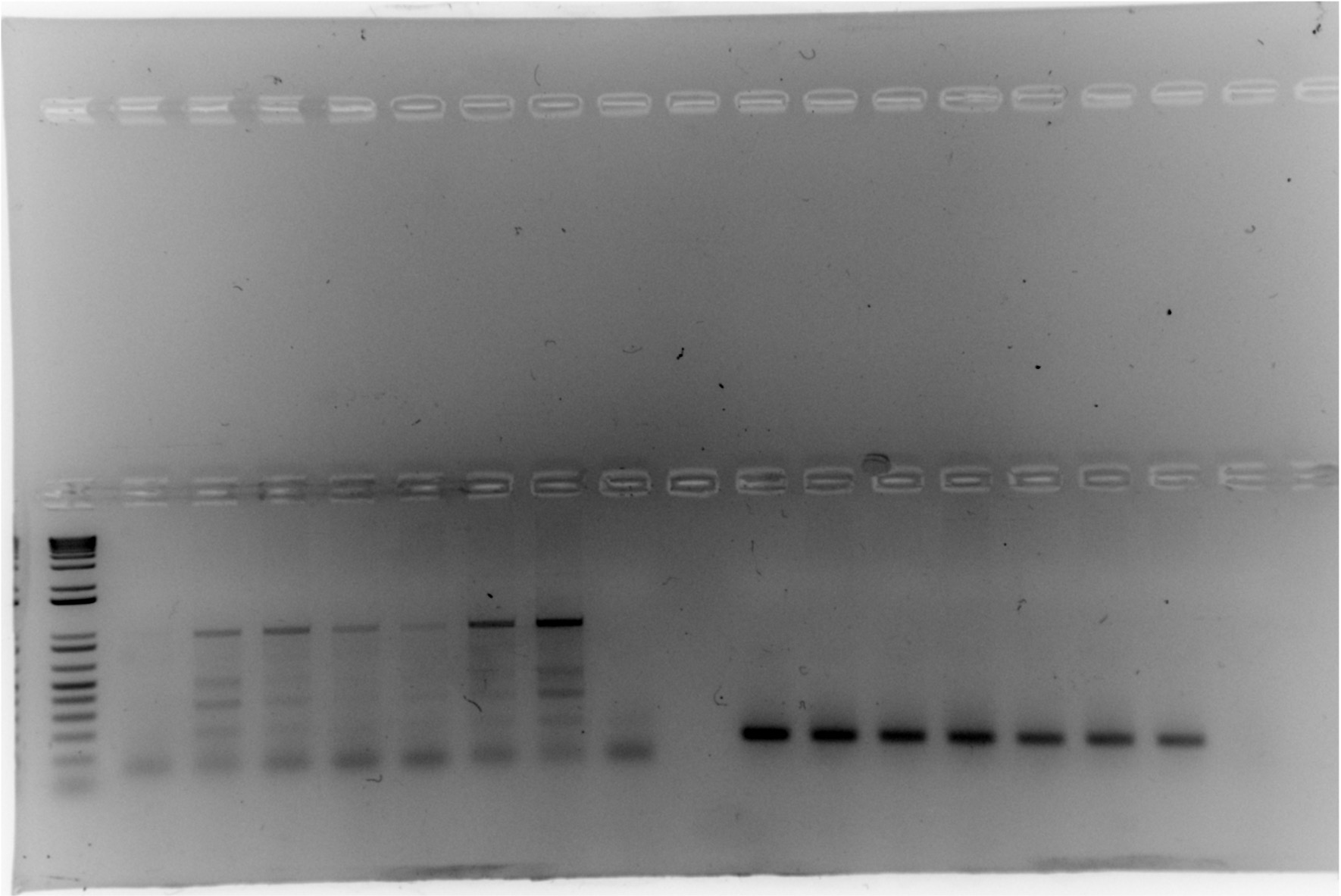

Supplement: Supplementary file 4 [file DataSheet1.zip › Right Arm and GAPDH.tif]
